# Nervous Fluid

**Published:** 1845-02

**Authors:** 


*Nervous Fluid.*—At a sitting of the Academy of Sciences of Paris, on the 10th of June last, MM. Thilorier and Lafontaine, communicated to the Academy that they had discovered a *new imponderable fluid*, resembling electricity or galvanism, but dissimilar from either.—*Ibid.*

